# Significant inter- and intra-laboratory variation in grading of ductal carcinoma in situ of the breast: a nationwide study of 4901 patients in the Netherlands

**DOI:** 10.1007/s10549-018-05082-y

**Published:** 2018-12-11

**Authors:** Carmen van Dooijeweert, Paul J. van Diest, Stefan M. Willems, Chantal C. H. J. Kuijpers, Lucy I. H. Overbeek, Ivette A. G. Deckers

**Affiliations:** 1 Department of Pathology, University Medical Centre Utrecht, PO Box 85500, 3508 GA Utrecht, The Netherlands; 2 Foundation PALGA (the nationwide network and registry of histo-and cytopathology in the Netherlands), Houten, The Netherlands

**Keywords:** DCIS, Ductal carcinoma in situ, Prognostic factor, Histologic grade, Pathology, PALGA

## Abstract

**Purpose:**

A considerable part of ductal carcinoma in situ (DCIS) lesions may never progress into invasive breast cancer. However, standard treatment consists of surgical excision. Trials aim to identify a subgroup of low-risk DCIS patients that can safely forgo surgical treatment based on histologic grade, which highlights the importance of accurate grading. Using real-life nationwide data, we aimed to create insight and awareness in grading variation of DCIS in daily clinical practice.

**Methods:**

All synoptic pathology reports of pure DCIS resection specimens between 2013 and 2016 were retrieved from PALGA, the nationwide Dutch Pathology Registry. Absolute differences in proportions of grade I-III were visualized using funnel plots. Multivariable analysis was performed by logistic regression to correct for case-mix, providing odds ratios and 95% confidence intervals for high-grade (III) versus low-grade (I–II) DCIS.

**Results:**

4952 DCIS reports from 36 laboratories were included, of which 12.5% were reported as grade I (range 6.1–24.4%), 39.5% as grade II (18.2–57.6%), and 48.0% as grade III (30.2–72.7%). After correction for case-mix, 14 laboratories (38.9%) reported a significantly lower (*n* = 4) or higher (*n* = 10) proportion of high-grade DCIS than the reference laboratory. Adjusted ORs (95%CI) ranged from 0.52 (0.31–0.87) to 3.83 (1.42–10.39). Significant grading differences were also observed among pathologists within laboratories.

**Conclusion:**

In this cohort of 4901 patients, we observed substantial inter- and intra-laboratory variation in DCIS grading, not explained by differences in case-mix. Therefore, there is an urgent need for nationwide standardization of grading practices, especially since the future management of DCIS may alter significantly depending on histologic grade.

**Electronic supplementary material:**

The online version of this article (10.1007/s10549-018-05082-y) contains supplementary material, which is available to authorized users.

## Introduction

Ductal carcinoma in situ (DCIS) of the breast is generally considered a precursor of invasive ductal carcinoma [1], although it is unknown which proportion of untreated DCIS lesions will progress into invasive breast cancer [2]. In fact, it is believed that a considerable part of DCIS patients is treated for lesions that may never progress into invasive breast cancer [3–5]. Nevertheless, standard treatment of DCIS currently consists of either mastectomy or breast-conserving surgery followed by radiotherapy and/or followed by endocrine therapy [6]. In addition, standard treatment decisions are made regardless of histologic grade, while progression risk or at least speed of progression is higher for high-grade lesions [7, 8].

To counteract this presumed overtreatment, four clinical trials currently aim to identify a subgroup of low-risk DCIS patients that, under active surveillance, could safely forgo surgical treatment [3, 4, 9, 10]. All these trials aim to identify this subgroup solely based on histologic grade, hence, histologic grading of DCIS might be of great clinical importance in the near future. This perspective highlights the key importance of accurate, consistent, and reproducible grading by pathologists. However, current evidence suggests that there is considerable variation in the grading of DCIS in daily clinical practice. Previous studies in which several pathologists reviewed a set of DCIS cased show that the classification of DCIS is associated with significant inter-observer variation [11–13]. Moreover, various classifications are used to subdivide DCIS into lesions of good (grade I), moderate (grade II), and poor (grade III) differentiation. Although previous studies showed that there is considerable variation in grading, individual practicing pathologists may not have been influenced by these data as it did not provide them insight into their own grading practice. Moreover, grading was performed in a study setting, which may not resemble grading in real-life clinical practice. In this context, studies in a nationwide cohort of (pre)malignant colorectal lesions in real-life daily clinical practice by Kuijpers et al. showed considerable inter-laboratory differences in the histologic grading of both colorectal adenomas and adenocarcinomas [14, 15]. Therefore, we expected substantial variation between pathology laboratories, as well as individual pathologists in the grading of breast DCIS in daily clinical practice. To create insight and awareness in grading variation of DCIS, especially with the potential future treatment consequences in mind, we studied the laboratory-specific variation in histologic grading of DCIS in a nationwide daily clinical practice study.

Using the Dutch nationwide pathology registry (PALGA), we assessed the variation in histologic grading of nearly 5000 patients with DCIS between Dutch pathology laboratories and between individual pathologists using data from synoptic (structured) pathology reports from real-life daily pathology practice. Furthermore, we conducted a questionnaire among pathologists to gain insight into their histologic grading practices. Creating insight into these laboratory-specific differences may help design an intervention to improve standardization among laboratories and pathologists. This may ultimately enable more accurate risk stratification of patients with low-risk DCIS, which is highly relevant since the future management of DCIS may alter significantly depending on histologic grade.

## Materials and methods

### Data source and study population

Data were extracted from the PALGA database, the nationwide registry of histo- and cytopathology in the Netherlands, which contains excerpts of all pathology reports from Dutch pathology laboratories since 1991 [16]. Data from the PALGA database are pseudonymised by a trusted third party (ZorgTTP, Houten, the Netherlands). All Dutch laboratories gave consent for the storage of their data in the PALGA database, including scientific use of these data. Additional consent was obtained for analysis of inter-pathologist variation within the individual laboratories. All laboratories were anonymized. The scientific and privacy committee of PALGA approved this study.

We retrieved all synoptic pathology reports of DCIS resection specimens without the presence of any coexistent invasive component between January 1, 2013 and December 31, 2016 in the Netherlands. Synchronous DCIS was defined as an ipsilateral DCIS lesion within six months of the first DCIS diagnosis. These lesions were considered paired measurements of which only the first was included. We excluded DCIS pathology reports of re-excisions and reports of residual in situ lesions after neoadjuvant treatment of primary invasive tumors.

In total, 39 out of 46 Dutch laboratories synoptically reported DCIS on breast resection specimens. Of these, we included those that synoptically reported ≥ 75 DCIS during the study period. Similarly, for inter-pathologist variation within individual laboratories, we analyzed only data from pathologists who synoptically reported ≥ 10 DCIS during the study period. Lastly, reports of DCIS with unknown tumor grade or unknown tumor size were excluded from further data analysis.

From each report, we extracted patients’ sex and age, DCIS size, histologic grade, type of surgery, and date of diagnosis.

### Histologic grading

The primary outcome measure of this study was the inter-laboratory variation in histologic grading of DCIS. DCIS grade III was considered as high grade, whereas DCIS grades I and II were considered low-grade in our multivariate inter-laboratory analysis. Secondary outcome measure was the inter-pathologist variation in histologic grading within the individual laboratories.

### Questionnaire among pathologists

A questionnaire was sent to all pathology laboratories in the Netherlands to identify how pathologists determine the histologic grade of DCIS in daily practice. The questionnaire contained questions on whether pathologists consider themselves as specialized breast pathologists, the number of years of experience as a pathologist, which classification system they use for grading DCIS, and how they deal with heterogeneity of histologic grade within one specimen.

### Statistical analysis

The overall proportions of histologic grades I, II, and III DCIS were determined and considered the national proportion. Absolute differences in proportion of histologic grades between laboratories are presented in funnel plots per grade, in which the proportions per laboratory were plotted against the number of included DCIS per laboratory. The target of these funnel plots was set at the national proportions with 95% confidence intervals (CI) as limits [17].

To compare relative differences among laboratories, odds ratios (OR) and 95% CIs per laboratory were calculated by logistic regression. For the choice of the reference laboratory, the sum-score of absolute deviations from the grade-specific national proportions was calculated to compare the absolute deviation for all three grades at once. The laboratory with the lowest sum-score was deemed best for resembling the national distribution and was therefore chosen as the reference laboratory.

Patient and tumor characteristics were summarized and differences between histologic grades were tested by means of a χ^2^-test for categorical variables and by a non-parametric Kruskal–Wallis test for continuous variables.

A multivariate logistic regression analysis was performed to correct for differences in case-mix. To identify these potential confounding factors, we selected clinicopathological variables a priori based on literature [18–20] and on pathologists’ experience, namely, age, sex, tumor size, type of surgery, and year of diagnosis. Only tumor size and type of surgery appeared to be significantly associated with grade and were therefore included in the final multivariate model. Since differences between the univariate and multivariate models were limited, only the adjusted ORs (95% CI) are presented in a forest plot.

For analysis of the inter-pathologist variation within the individual laboratories, we merely compared the proportions per histologic grade between pathologists by Fisher exact test. Results of the questionnaire were summarized by frequencies and percentages.

*P* values below 0.05 were considered statistically significant. All statistical analyses were performed by using IBM SPSS Statistics version 21.

## Results

### Characteristics of patients, DCIS lesions, and laboratories

**Fig. 1:**
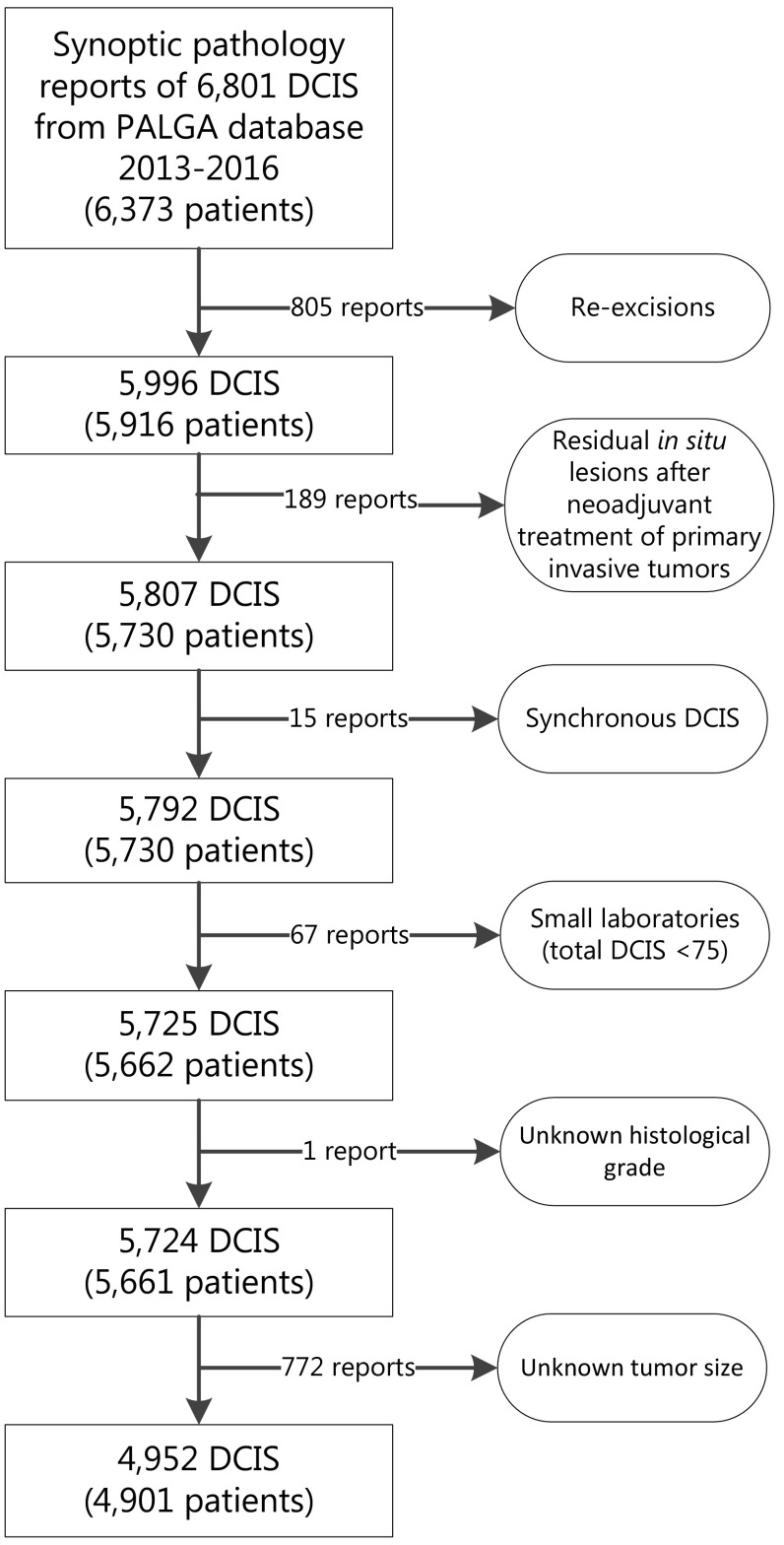
Flowchart of included cases of ductal carcinoma in situ (DCIS) of the breast to assess histopathologic grading variation between laboratories

A total of 4952 DCIS lesions of 4901 patients from 36 laboratories were included in our final data analysis (Fig. 1). Characteristics of these included patients and corresponding DCIS are listed in Table 1. Mean (SD) age at diagnosis was 59.5 (10.1) years and patients were predominantly female (99.8%). The majority of patients underwent breast-conserving surgery (67.0%). Both tumor size and mastectomy rate increased with histologic grade.

**Table 1:** Characteristics of the 4952 included cases of ductal carcinoma in situ (DCIS) of the breast

	Total (*n* = 4592)	Grade 1 (*n* = 618)	Grade 2 (*n* = 1956)	Grade 3 (*n* = 2378)	p-value
Age (years)^a^	59.5 (10.1)	58.5 (10.2)	59.9 (10.1)	59.3 (10.1)	0.001
Sex, *n* (%)					^b^
Female	4944 (99.8%)	616 (99.7%)	1950 (99.7%)	2378 (100.0%)	
Male	8 (0.2%)	2 (0.3%)	6 (0.3%)	0 (0.0%)	
Tumor size (cm)*	2.4 (2.2)	1.6 (1.8)	2.1 (2.0)	2.9 (2.3)	0.000
Type of surgery, *n* (%)					0.000
Mastectomy	1636 (33.0%)	130 (21.0%)	538 (27.5%)	968 (40.7%)	
Breast conserving	3316 (67.0%)	488 (79.0%)	1418 (72.5%)	1410 (59.3%)	
Year of diagnosis, *n* (%)					0.518
2013	1051 (21.2%)	125 (20.2%)	438 (22.4%)	488 (20.5%)	
2014	1157 (23.4%)	147 (23.8%)	447 (22.9%)	563 (23.7%)	
2015	1298 (26.2%)	161 (26.1%)	489 (25.0%)	648 (27.2%)	
2016	1446 (29.2%)	185 (29.9%)	582 (29.8%)	679 (28.6%)	

^a^Mean (SD)

^b^Number of males too low for statistical testing

The number of synoptically reported DCIS lesions per laboratory ranged from 22 to 324 (median 109). Overall national proportions for DCIS grades I, II, and III were 12.5%, 39.5%, and 48.0%, respectively.

### Inter-laboratory differences in histologic grading

**Fig. 2:**
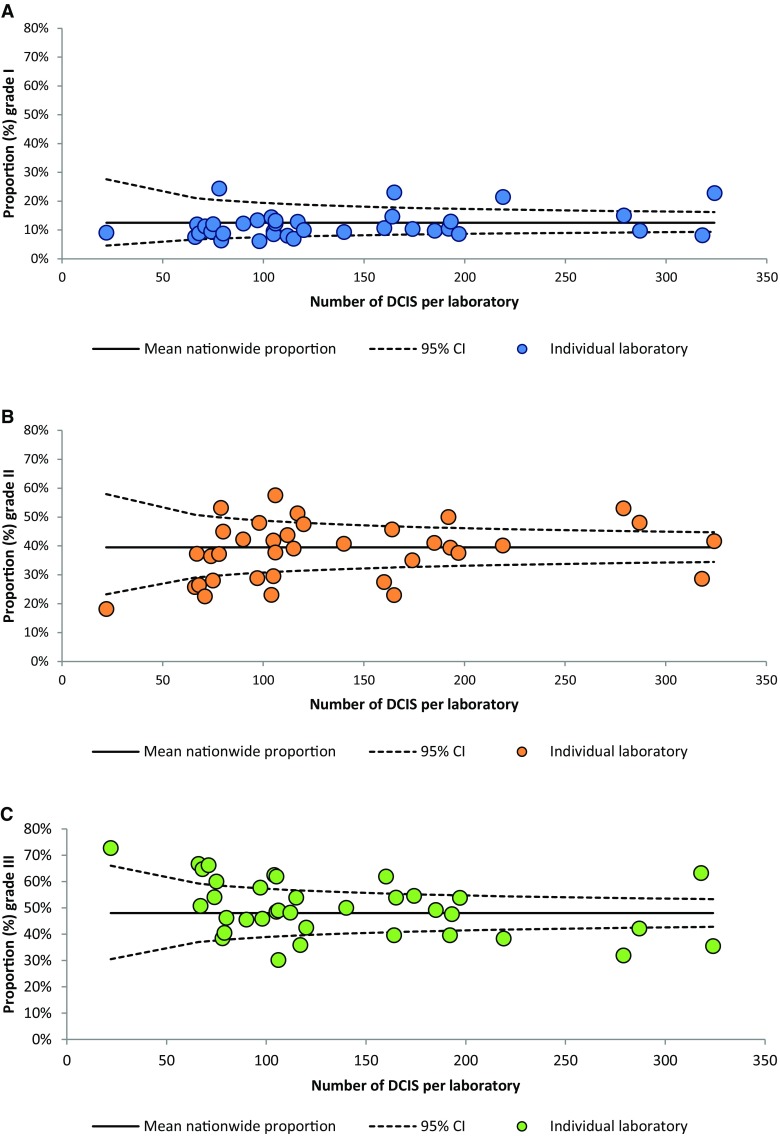
Funnel plots showing the observed proportion of ductal carcinoma in situ (DCIS) of the breast per grade per laboratory (dots) relative to the national proportion and its 95% confidence intervals for DCIS grades I (**a**), II (**b**), and III (**c**) (2013–2016)

Laboratories varied mostly in DCIS grade III (range 30.2–72.7%), followed by DCIS grade II (range 18.2–57.6%), and DCIS grade I (range 6.1–24.4%). Overall, half of the laboratories (18/36) showed proportions outside the 95% CI for grade III, indicating that these laboratories graded significantly different from the national proportion, which was similar for grade II (17 laboratories). In contrast, for grade I, only eight laboratories graded significantly different from the national proportion (Fig. 2).

**Fig. 3:**
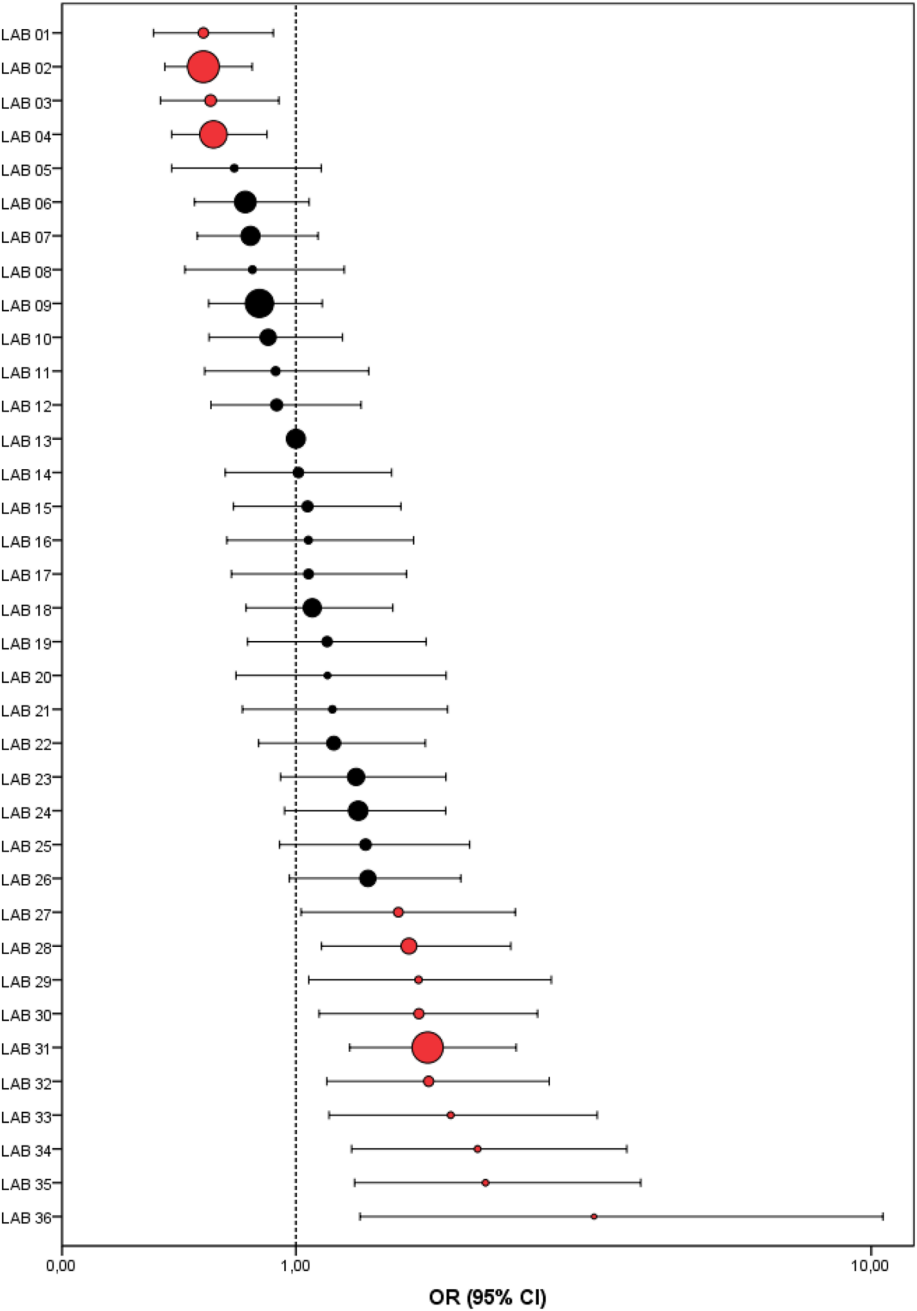
Forest plot showing the adjusted odds ratios (OR) and 95% confidence intervals (CI) of high-grade ductal carcinoma in situ (DCIS) of the breast (grade III) versus low-grade DCIS (grade I–II) per laboratory in comparison to the reference laboratory (#13). Dot size indicates the total number of analyzed synoptically reported DCIS cases per laboratory. Red dots indicate laboratories with a significantly deviant OR as compared to the reference laboratory

The sum-score was lowest and only 0.95% for laboratory 13, which was therefore chosen as reference laboratory. The maximum sum-score, in contrast, was 49.4%. Multivariate logistic regression showed that 14 laboratories (38.9%) reported a significantly higher (*n* = 10) or lower (*n* = 4) proportion of high-grade DCIS than the reference laboratory (Fig. 3). For two laboratories (laboratories 27 and 29), the conclusion on multivariate analysis differed from the conclusion on univariate logistic regression analysis. The ORs of these two laboratories became significantly deviant. Adjusted ORs (95% CI) of individual laboratories for high- versus low-grade DCIS ranged from 0.52 (0.31–0.87) to 3.83 (1.42–10.39).

### Intra-laboratory differences in histologic grading

**Fig. 4:**
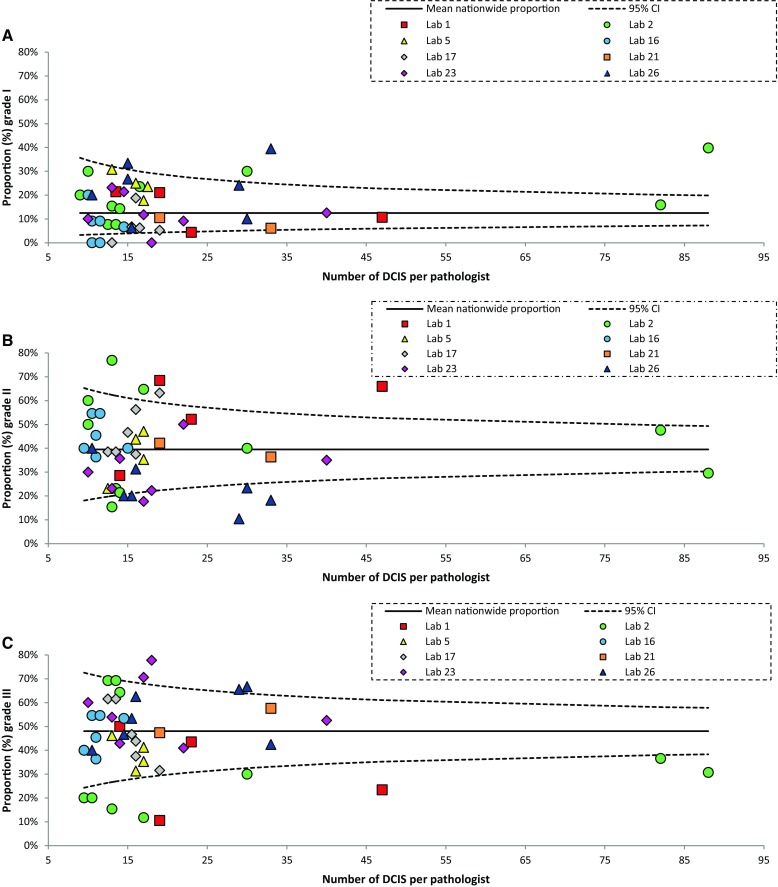
Funnel plots showing the observed proportion of ductal carcinoma in situ (DCIS) of the breast per grade per pathologist (dots) of eight laboratories relative to the national proportion for DCIS grades I (**a**), II (**b**) and III (**c**) (2013–2016)

Forty-six pathologists from eight analyzed laboratories synoptically reported ≥ 10 DCIS during the study period. Per laboratory, the number of analyzed pathologists ranged from 2 to 10 (median 6). In addition, the number of analyzed DCIS per pathologist ranged from 10 to 88 (median 15.5). Overall, 14 pathologists (28.3%) graded significantly deviant compared to the national proportions for grade II and III DCIS, while this was the case for ten pathologists (21.7%) for grade I (Fig. 4). Together, pathologists within individual laboratories differed mostly in the reporting of grade II (range 15.4–76.9%) and grade III (11.8–69.2%), both in laboratory 2. In contrast, the maximum variation for DCIS grade I was considerably smaller (range 6.3–39.4%) and was found in laboratory 26.

The differences in the distribution of histologic grade of DCIS among pathologists within the laboratories were significant in two laboratories (laboratories 1 and 2; Supplementary Fig. 1).

### Results of questionnaire

Seventy-nine (25%) pathologists (out of the approximately 320 practicing pathologists in the Netherlands [14]) responded to our online questionnaire, of which 37 (46.8%) were reportedly specialized breast pathologists. Grading practice of general and specialized breast pathologists did not seem to differ. Pathologists reported numerous different guidelines, articles, and books as a reference for the histologic grading of DCIS, with most pathologists (35.4%) using the guideline of Holland et al. [21]. Sixteen pathologists (20.3%) stated that they (partially) grade DCIS based on intuition. The majority of pathologists (76.0%) graded DCIS of heterogeneous differentiation based on the highest grade (Table 2).

**Table 2:** Results of 79 pathologists responding to our questionnaire on histologic grading of ductal carcinoma in situ (DCIS) of the breast

	*n* (%)		
	Total (*n* = 79)	Breast pathologist (*n* = 37)	General pathologist (*n* = 42)
Laboratory			
Academic	15 (19.0%)	11 (29.7%)	4 (9.5%)
Peripheral	64 (81.0%)	26 (70.3%)	38 (90.5%)
Years of experience			
0–5	28 (35.4%)	11 (29.7%)	17 (40.5%)
6–10	15 (19.0%)	6 (16.2%)	9 (21.4%)
11–20	17 (21.5%)	9 (24.3%)	8 (19.0%)
>20	19 (24.1%)	11 (29.7%)	8 (19.0%)
Based on which guideline or reference do you grade DCIS?^a^			
Holland et al. [21]	28 (35.4%)	14 (37.8%)	14 (33.3%)
Pinder et al. [22]	16 (20.3%)	9 (24.3%)	7 (16.7%)
Intuition	16 (20.3%)	8 (21.6%)	8 (19.0%)
WHO [23]	11 (13.9%)	5 (13.5%)	6 (14.3%)
I do not know	4 (5.1%)	0 (0.0%)	4 (9.5%)
Tavassoli et al. [24]	3 (3.8%)	1 (2.7%)	2 (4.8%)
Van Nuys (Silverstein et al. [25])	2 (2.5%)	1 (2.7%)	1 (2.4%)
College of American Pathologists Guidelines [26]	1 (1.3%)	0 (0.0%)	1 (2.4%)
Combination (n.o.s.)	1 (1.3%)	1 (2.7%)	0 (0.0%)
How do you grade a DCIS of heterogeneous differentiation?			
Based on the highest grade	60 (76.0%)	28 (75.7%)	32 (76.2%)
I report the percentages of each grade	9 (11.4%)	4 (10.8%)	5 (11.9%)
Based on the predominant grade	7 (8.9%)	4 (10.8%)	3 (7.1%)
Not within the protocol	3 (3.8%)	1 (2.7%)	2 (4.8%)

*n.o.s*. not otherwise specified

^a^Multiple answers possible (*n* = 82)

## Discussion

Using real-life daily clinical practice data from the nationwide pathology database PALGA, we studied the inter-laboratory variation in histopathologic grading of DCIS in daily clinical practice to create insight and awareness in grading variation, which is highly relevant as the future management of DCIS may alter significantly depending on histologic grade.

Approximately, half of the lesions of this nationwide cohort of 4952 DCIS were reported as grade III (48.0%), whereas 39.5% were reported as grade II and only 12.5% as grade I. The observed overall proportions per grade in this study are in line with previous studies of smaller cohorts of DCIS patients (*n* = 853–1430), which showed similar distribution percentages for DCIS grades I (15–18%), II (32–39%), and III (42–54%) [19].

Laboratory-specific data were analyzed in an absolute and relative manner, comparing individual laboratories to both the national proportion and the reference laboratory. This indicates that inter-laboratory differences in histologic grading were substantial. This was highlighted by the substantial range of absolute proportions of histologic grade between laboratories, by the sum-score that varied up to 48.5%, by the number of laboratories with significantly deviant proportions from the national distribution per grade (~ 50% for grade II and III), and by the number of laboratories with significantly deviant case-mix adjusted ORs from the reference laboratory (~ 40%). Case-mix adjusted ORs (95%CI) ranged between 0.52 (0.31–0.87) and 3.83 (1.42–10.39), indicating that the chance of a DCIS being graded as high grade in the ‘lowest’ laboratory is approximately two times lower than in the reference laboratory, and nearly four times higher in the ‘highest’ laboratory. Consequently, the difference between the ‘lowest’ and the ‘highest’ laboratory is even larger.

Although unlikely, since breast case-mix is not known to show regional differences in the Netherlands, we could not a priori exclude the possibility that grading practices of DCIS lesions may be influenced by patient and tumor characteristics. Comparison of the results of univariate and multivariate regression analysis, however, showed that the substantial inter-laboratory variation is not influenced by the most important clinicopathologic variables. Nonetheless, other variables, like imaging or how the lesions were diagnosed could play a role. Unfortunately, these factors are not, or very rarely, documented in pathology reports. However, the Dutch breast cancer-screening program refers patients randomly to all local Dutch hospitals and it is therefore unlikely to be an important factor in case-mix correction. This is further supported by the small effect of the case-mix variables that could be taken into account in the current study, which implies that regional differences in breast case-mix are limited in the Netherlands. Another possible confounder that could not be analyzed is comedo necrosis, since this is not an obligatory variable in the synoptic reporting module and was only available in a minority of cases. We have, however, no reason to assume that this feature is not evenly distributed between laboratories. Lastly, all laboratories were anonymized to the researchers, but we do know that five of the eight Dutch academic laboratories are represented in our dataset. There were no striking differences between academic and regional laboratories, but the incomplete dataset does not allow to draw firm conclusions.

For multivariable data analysis, histologic grade was dichotomized into low-grade DCIS (grade I–II) and high-grade DCIS (grade III), based on the definition of low-risk DCIS in the majority of current clinical trials [3, 4, 9, 10]. Moreover, given the low proportion of grade I DCIS, most variation between laboratories was expected between grade II and III. In a sensitivity analysis, we were able to validate the main results of our logistic regression model in a multinomial regression model, which allows a multinomial endpoint (data not shown).

Data included in this study were merely from patients synoptically reported DCIS lesions, because synoptic reporting, compared to narrative reporting, results in improved reporting of relevant clinical data and an increased overall completeness of pathology reports [27]. In addition, all variables are stored in a standardized manner, which enables reliable and easy data extraction. Over 80% of (pre)malignant breast lesions is currently reported synoptically by pathologists via the PALGA protocol [28]. To check whether our case selection was likely to be representative for all DCIS patients, we compared our data with aggregated data from the Netherlands Cancer Registry, which also holds narrative reports, and observed a similar distribution of histologic grade (data not shown), indicating that our case selection based on synoptic reporting is likely to be representative.

Because we used data from a nationwide pathology database, we were able to extract reports of pure DCIS lesions, indicating that reports did not indicate the presence of any coexistent invasive component. This is important because grading of DCIS lesions may not be independent of the invasive component. For example, Farabegoli et al. showed that pure DCIS and DCIS associated with invasive ductal carcinoma may be genetically distinct [29]. It is unclear whether a previous history of invasive breast cancer might influence the grading of a later DCIS lesion, but it is unlikely that a pathologist being aware of the breast pathology history would interpret DCIS morphology differently. Nevertheless, the influence on our results would be limited as the proportion of patients with such history in our dataset was small (~ 5%).

In addition to the substantial inter-laboratory variation, significant differences were also observed between pathologists within two out of eight analyzed laboratories. These results emphasize that even within the laboratories analyzed, histologic grading is not performed in a standardized manner. Moreover, this implies that the observed inter-laboratory variation may predominantly be the result of different grading practices of individual pathologists and not of differences in case-mix, as shown in the present study. The findings from our questionnaire, where numerous reference classifications were mentioned as a guideline by pathologists and 20% of pathologists even stated that they grade DCIS based on intuition, further illustrate that histologic grading is currently insufficiently standardized. This calls for (inter)national consensus on the grading system and criteria to improve reproducibility in view of the therapeutic consequences. About half the intuitive graders regarded themselves as breast pathologists, which calls for better criteria for this status.

The results of this study may raise awareness among pathologists, emphasizing that histologic grading of DCIS is currently not meeting high enough standards, which is an important first step to improvement. The fact that we needed a threshold of 10 to allow analysis of inter-pathologist variation indicates that perhaps too many pathologists engage in DCIS diagnosis. In addition, pathologists are enabled to discuss and reflect on their grading practices, as these “mirror” data were also sent to the laboratories by PALGA. In this context, annual benchmarking of histologic grading of DCIS based on “mirror” PALGA data is already being considered by the Dutch Society for Pathology and may be adopted much broader in the field. Future research might focus on the development of an e-learning module to train pathologists in determining the histologic grade of DCIS, thereby aiming to attribute to the synchronization and better reproducibility of DCIS grading.

This improvement is especially relevant since the decision to manage low-risk DCIS through active surveillance may be solely dependent on histologic grade in the near future. However, the risk stratification of all four clinical trials [3, 4, 9, 10] based merely on histological grade is criticized by Toss et al. [7], who emphasize that trial outcomes will be influenced by the inherent subjectivity of the current simple grading system and that DCIS risk stratification should be a combination of histologic grading and more objective biomarkers such as ER and HER2 [7], molecular markers, or deep learning strategies on digital images. In this context, standardization and synchronization of histologic grading will not only improve health care in general, but it might also improve risk stratification of low-risk DCIS and subsequently might improve (clinical) studies that take histologic grading into account.

In conclusion, both inter- and intra-laboratory results of this nationwide cohort of nearly 5000 patients show that there is substantial variation in the histologic grading of DCIS by pathologists in routine daily clinical practice. This implies that there is an urgent need for improvement and better standardization of DCIS grading, especially since the future management of DCIS may alter significantly depending on histologic grade.

## Electronic supplementary material

Below is the link to the electronic supplementary material.

Supplementary material 1 (DOCX 94 KB)

Supplementary material 2 (DOCX 92 KB)
