# Supplementary material for: Significant inter- and intra-laboratory variation in grading of ductal carcinoma in situ of the breast: a nationwide study of 4901 patients in the Netherlands
Source: Breast Cancer Res Treat. 2018 Dec 11;174(2):479–88. doi: 10.1007/s10549-018-05082-y (PMC6422994; doi:10.1007/s10549-018-05082-y)
Supplement: Supplementary file 2 — Supplementary material 2 (DOCX 92 KB) [file 10549_2018_5082_MOESM2_ESM.docx]

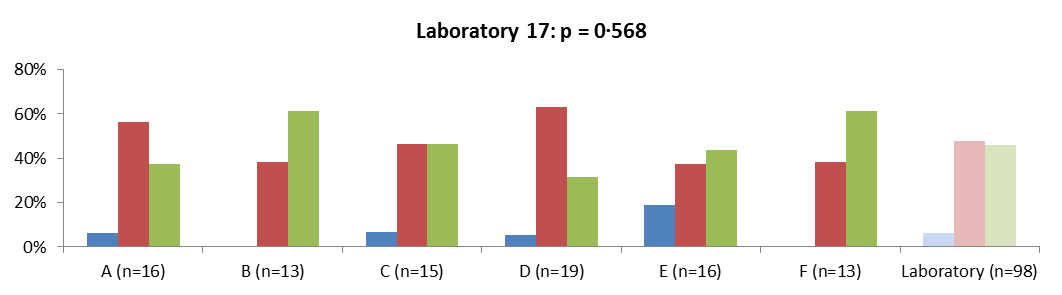

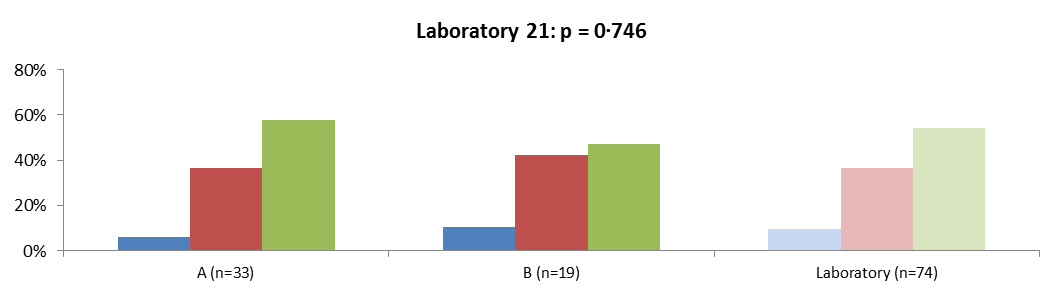

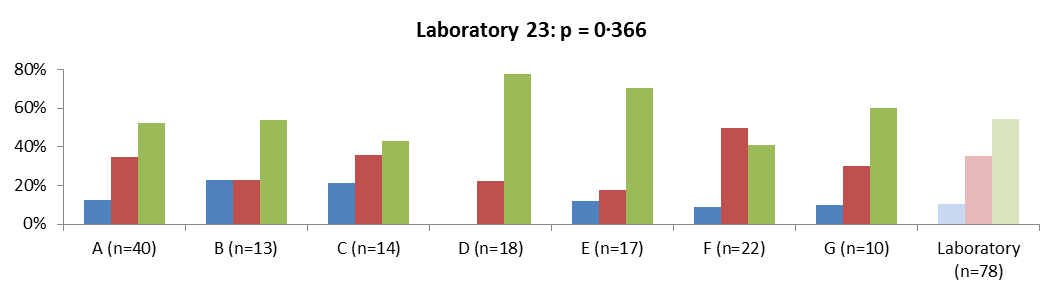

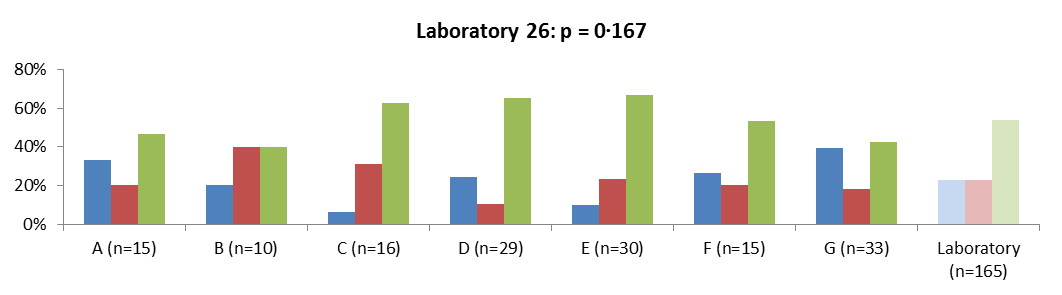


SUPPLEMENTARY 1B. Intra-laboratory variation between pathologists in laboratories 17, 21, 23 and 26. Blue, red and green bars indicate the proportions of ductal carcinoma *in situ* (DCIS) grades I, II and III, respectively. Capital letters on the X-axis indicate pathologists within one laboratory, followed by the overall proportion of the laboratory per differentiation grade. Intra-laboratory differences are calculated by Fisher exact test (Monte Carlo option in laboratory 26).
